# A new approach to treatment of resistant gram-positive infections: potential impact of targeted IV to oral switch on length of stay

**DOI:** 10.1186/1471-2334-6-94

**Published:** 2006-06-08

**Authors:** Mohammed Desai, Bryony Dean Franklin, Alison H Holmes, Sarah Trust, Mike Richards, Ann Jacklin, Kathleen B Bamford

**Affiliations:** 1 Department of Infectious Diseases and Microbiology, Imperial College, London, UK; 2 Pharmacy Department, Hammersmith Hospitals NHS Trust, London, UK; 3 Department of Microbiology, Hammersmith Hospitals NHS Trust, London, UK; 4 Department of Infectious Diseases, Hammersmith Hospitals NHS Trust, London, UK; 5 The School of Pharmacy, University of London, London, UK

## Abstract

**Background:**

Patients prescribed intravenous (IV) glycopeptides usually remain in hospital until completion of this treatment. Some of these patients could be discharged earlier if a switch to an oral antibiotic was made. This study was designed to identify the percentage of inpatients currently prescribed IV glycopeptides who could be discharged earlier if a switch to an oral agent was used, and to estimate the number of bed days that could be saved. We also aimed to identify the patient group(s) most likely to benefit, and to estimate the number of days of IV therapy that could be prevented in patients who remained in hospital.

**Methods:**

Patients were included if they were prescribed an IV glycopeptide for 5 days or more. Predetermined IV to oral antibiotic switch criteria and discharge criteria were applied. A multiple logistic regression model was used to identify the characteristics of the patients most likely to be suitable for earlier discharge.

**Results:**

Of 211 patients, 62 (29%) could have had a reduced length of stay if they were treated with a suitable oral antibiotic. This would have saved a total of 649 inpatient days (median 5 per patient; range 1–54). A further 31 patients (15%) could have switched to oral therapy as an inpatient thus avoiding IV line use. The patients most likely to be suitable for early discharge were those with skin and soft tissue infection, under the cardiology, cardiothoracic surgery, orthopaedics, general medical, plastic surgery and vascular specialities, with no high risk comorbidity and less than five other regularly prescribed drugs.

**Conclusion:**

The need for glycopeptide therapy has a significant impact on length of stay. Effective targeting of oral antimicrobials could reduce the need for IV access, allow outpatient treatment and thus reduce the length of stay in patients with infections caused by antibiotic resistant gram-positive bacteria.

## Background

The incidence of infection with resistant gram-positive organisms has increased dramatically over the past 10 years [1]. In the UK, this relates mainly to hospital-acquired infections. However, there are also serious worldwide concerns about community-acquired infections with resistant organisms [2,3]. Methicillin resistant *Staphylococcus aureus *(MRSA) is the single most important contributor to antimicrobial resistant nosocomial infections [4].

One consequence of the increase in antimicrobial resistance among gram-positive bacteria is an increase in the use of glycopeptides. These generally require intravenous (IV) administration, resulting in an increase in the use of IV lines. This in turn results in reduced mobility, and increased risk of line-related infections. Presence of IV lines is the most significant risk factor for hospital-acquired bacteraemia and endocarditis [5,6]. Use of vancomycin, the most widely used glycopeptide, is also associated with nephrotoxicity [7,8] and requires individualised dosing and monitoring of serum levels. Except in rare examples of effective home IV programmes [9], glycopeptide treatment inevitably means that the individual must stay in hospital. There are well-recognised adverse consequences of IV line use and hospitalisation, particularly in older and more seriously ill patients [10]. In contrast, many infections caused by methicillin sensitive *S. aureus *can be treated effectively with oral antibiotics, or a few days of IV antibiotics followed by oral therapy for the remainder of the course. Where a suitable oral agent exists, IV to oral switch programmes have been shown to be highly effective [11-13].

Until recently, there have been no effective single oral agents for the treatment of many resistant gram-positive infections, and there are no measures of the likely impact on length of stay if a suitable agent was to become available. However, linezolid, an antimicrobial with activity against MRSA that can be given orally, was licensed in the UK in 2001 and is at least as effective as glycopeptides [14,15]. Unfortunately however, linezolid is significantly more expensive than glycopeptides, and there are concerns that widespread use may lead to the development of resistance [16]. It may therefore be inappropriate to use linezolid in all inpatients with resistant gram-positive infections, so targeting those patients most likely to benefit by being discharged earlier may be the most appropriate approach.

The aims of this study were to explore the potential impact of substituting an equally effective oral agent for IV glycopeptides on IV line use and length of stay, and to identify the patient groups most likely to benefit. Specific objectives were:

• to identify the percentage of patients currently prescribed IV glycopeptides who could be discharged earlier if an oral agent was used;

• to estimate the number of inpatient days that could be saved;

• to identify the groups of patients most likely to be suitable for earlier discharge if an oral agent was used;

• to identify the additional number of days of IV antibiotic treatment that could be prevented by using an oral agent in hospitalised patients.

## Methods

### Setting

The study took place in a 1026 bed NHS teaching hospital trust in west London, on two main hospital sites, and across a broad range of general, regional and tertiary referral specialties. In accordance with the trust's antibiotic guidelines, vancomycin was used as the first line glycopeptide. Guidelines suggested the most appropriate initial dose based on age, weight and renal function; these also advised that trough levels be taken before the third or fourth dose and twice weekly thereafter. Teicoplanin was approved only for the management of febrile neutropaenia or where vancomycin was contraindicated or not tolerated. Each ward was allocated a pharmacist who visited the ward each weekday to check that all medication orders were legal, clear and clinically appropriate for the patient.

The study was approved by the Hammersmith, Queen Charlotte's and Acton Hospitals Research Ethics Committee.

### Patient recruitment

Following development and piloting of the methods and data collection processes, ward pharmacists in the two hospital sites were asked to notify the study pharmacist (ST) of all inpatients prescribed vancomycin or teicoplanin during a six-month period beginning February 2002. This was augmented by daily visits by the study pharmacist to clinical areas with the highest use of glycopeptides, according to previous point-prevalence surveys of anti-infective use in our trust [17].

The study pharmacist and either a microbiologist or Infectious Diseases physician evaluated each patient after 48 hours of glycopeptide therapy. Patients were included in the study if they fulfilled the following criteria:

1. Suspected or proven gram positive infection;

2. Expected to need glycopeptide treatment for at least 5 days as judged by the relevant medical or surgical team;

3. Patient expected to survive with antibiotic therapy and supportive care;

4. None of the following exclusion criteria:

4.1. Suspected or proven left-sided endocarditis, osteomyelitis or prosthetic infection where the prosthesis could not be removed;

4.2. Per-protocol prescribing in haematology (where teicoplanin was prescribed in response to failure of fever resolution in neutropaenic patients without microbiological or clinical evidence of gram positive infection);

4.3. Aged less than 16 years;

4.4. Pregnant or lactating female;

4.5. Patients with any other contraindication to linezolid.

### Data collection

Patients meeting the inclusion criteria were assessed on alternate days by the study pharmacist until the end of glycopeptide treatment, discharge or death, whichever was sooner, to find out if and when they met predetermined criteria for either IV to oral switch, or discharge. Patients who were enrolled but in whom it became apparent that glycopeptide therapy was inappropriate, or who required less than 5 days glycopeptide treatment for any reason other than an unwanted reaction, were withdrawn from the study.

The IV to oral switch criteria (tables 1 and 2) were adapted from those already used in the trust [11] and elsewhere [12,13]. The date on which each patient first fulfilled these criteria was taken to be the date of theoretical IV to oral switch.

**Table 1:** IV to oral switch inclusion criteria used

**1. Clinical status**	• **Temperature less than 38°C for 24 hours** • **White cell count normalising** • **No unexplained tachycardia (Heart rate less than 100 beats per minute)** • **Sensitivity received (if microbiology positive)**
**2. Oral absorption**	• **Patient tolerates oral fluids** • **No medical problems leading to reduced oral absorption (e. g. vomiting, diarrhoea, and gastrointestinal surgery)** • **No surgical operation scheduled within next 36 hours**

**Table 2:** IV to oral switch exclusion criteria used

**1. Continuing sepsis**	• **Temperature less than 36°C or more than 38°C** • **White cell count less than 4 × 10^9^/L or more than 12 × 10^9^/L** • **Unexplained tachycardia (Heart rate greater than 100 beats per minute in last 12 hours)**
**2. Oral route compromised**	• **Vomiting or severe diarrhoea** • **Other ongoing or potential absorption problem**

Patients were graded as suitable for discharge on oral therapy if, after fulfilling the IV-oral switch criteria, they then fulfilled the discharge criteria (Table 3).

**Table 3:** Discharge on oral therapy criteria

**1. Clinical status**	• **Afebrile for at least 48 hours** • **Tolerates normal diet** • **IV access only required for glycopeptide therapy** • **Physically independent/well enough for residential/nursing home care**
**2. Clinical team discharge policy**	• **A YES answer to the question: "If this patient was on an equally effective oral antibiotic, could they be discharged today?"**

### Data management and analysis

Data collection forms were used to capture the information, which was anonymised and entered into a database (Microsoft Access). A logistic regression analysis was used to explore the characteristics of the patients most likely to benefit from earlier discharge. Analysis was performed using Genstat 5 (version 3.2; VSN International, Hemel Hempstead, UK).

## Results

A total of 211 patients fulfilled the study criteria, of whom 80 (38%) were female. Of these, 183 (87%) were prescribed vancomycin as the sole glycopeptide, and 20 (9%) teicoplanin alone. Eight patients (4%) received both drugs sequentially during the data collection period. Of these eight, six were changed from vancomycin to teicoplanin because of adverse effects, and two were started on teicoplanin but then changed to vancomycin in accordance with the trust's antibiotic policy. Adverse effects associated with glycopeptide use were identified in 8 (4%) patients. These were rash (4 patients), red man syndrome (1), cellulitis secondary to IV line (1), extravasation (1) and acute renal failure (1).

A total of 62 (29%) patients fulfilled the criteria for both IV to oral switch and discharge on oral therapy during the period they were prescribed an IV glycopeptide. For these 62 patients, 649 in-patient days could potentially have been saved during the 6-month study period (median 5 per patient, range 1–54). In addition, a further 31 patients (15%) fulfilled the IV to oral switch criteria but were not suitable for discharge. For these patients, the length of time they required IV therapy during their hospital stay could have therefore been reduced and their IV lines taken out earlier. An additional 247 days of IV therapy could have been saved in these patients, representing a median of 6 per patient (range 1–23).

The characteristics of the patients studied and their associations with suitability for early discharge or oral switch are shown (Tables 4, 5, and 6). Nearly all the patients deemed suitable for early discharge had infections of skin and soft tissue. By confining attention to patients with infection in this site (118 of the 211; 56%), it was found that five of the eleven clinical specialities had no patients suitable for early discharge; these were gastroenterology, haematology/oncology, neurology, other surgical and respiratory. For patients under the remaining specialities (100 of the 118 patients; 85%), multiple logistic regression models for suitability for early discharge were tried against infecting organism, number of comorbidities, presence of a high risk comorbidity (atrial fibrillation, cancer, immobility, or insulin dependent diabetes mellitus), number of other drugs (both as a quantitative variate and dichotomised as less than 5 or 5 or more), and speciality. A model with all these terms had deviance 19.9 on 13 degrees of freedom. A model with only presence of a high risk comorbidity, and five or more other drugs, had deviance 15.4 on 2 degrees of freedom (p < 0.001). No other terms were significant in the presence of these two.

**Table 4:** Patients studied – site of infection

**Site of infection**	**Total no. of patients in this category**	**Number of patients suitable for switch to oral therapy (% of patients with this site of infection)**	**Number of patients suitable for switch to oral therapy and early discharge (% of patients with this site of infection)**
**Blood**	40	8 (20%)	3 (8%)
**Sputum**	40	2 (5%)	2 (5%)
**Skin/Soft tissue**	118	77 (65%)	54 (46%)
**Other**	13	6 (46%)	3 (23%)

Total	211	93	62

**Table 5:** Patients studied – site of infection

**Infecting organism**	**Total no. of patients in this category**	**Number of patients suitable for switch to oral therapy (% of patients with this infecting organism)**	**Number of patients suitable for switch to oral therapy and early discharge (% of patients with this infecting organism)**
**Coagulase negative staph**	44	13 (30%)	9 (21%)
**Enterococcus**	17	5 (29%)	4 (24%)
**MRSA**	110	63 (57%)	38 (36%)
**Other organisms**	7	3 (43%)	3 (43%)
**Culture negative**	33	9 (27%)	8 (24%)

	Total = 211	93	62

**Table 6:** Characteristics of patients studied – Number of other drugs taken by patients.

**Number of other drugs prescribed**	**Number of patients prescribed this no. of other drugs**	**Number (%) patients in category suitable for switch to oral therapy**	**Number (%) patients in category suitable for switch to oral therapy and early discharge**
**1**	18	16 (89%)	10 (56%)
**2**	24	13 (54%)	5 (21%)
**3**	58	29 (50%)	25 (43%)
**4**	46	20 (44%)	15 (43%)
**5**	30	9 (30%)	5 (17%)
**6**	18	4 (22%)	1 (6%)
**7**	6	1 (17%)	1 (17%)
**8**	8	1 (13%)	0
**9**	1	0	0
**10**	2	0	0

	Total = 211	93 (44%)	62 (29%)

The patients most likely to be suitable for early discharge were therefore those with skin and soft tissue infection, under the cardiology, cardiothoracic surgery, orthopaedics, general medical, plastic surgery and vascular specialities, with no high risk comorbidity and who are prescribed less than five other regular drugs. In this data set, this identified 55 patients, 39 (71%) of whom were deemed suitable for early discharge. The speciality with the highest number of identified patients was orthopaedics, with 20 patients of whom 15 (75%) were deemed suitable for early discharge.

## Discussion

The availability of effective oral agents that can treat infections caused by resistant gram-positive organisms could have a significant impact on the management of the patients affected. The potential for hospital directed outpatient oral therapy has three possible benefits. First, the individual may be treated outside a hospital setting thus releasing in-patient resources for reallocation. Second, in the case of antibiotic resistant pathogens, the potential for transmission within the health care setting is reduced. Thirdly, the removal of IV lines reduces risk factors for hospital-acquired bacteraemia, endocarditis and phlebitis. While accurate financial accounting was not possible as part of this study we have reported the number of bed days saved as a marker of potential cost savings. The savings realised from a bed day saved needs to be balanced against the cost of outpatient oral antibiotic treatment and clinic follow up. However we still envisage substantial savings with this approach.

One retrospective evaluation of the potential to switch to linezolid has been carried out in Los Angeles, where 103 (58%) of 177 vancomycin courses could potentially have been switched to oral linezolid [18]. In our study, which was prospective, this figure was slightly less, at 45%. In addition, 53% of the 103 Los Angeles patients were potentially eligible for early discharge [18]. Again, this supports our findings. What may differ is that because there are variations in discharge and provision of effective home IV services there is potentially more benefit to patients in the UK and areas such as west London. In our study there was a smaller overall proportion of patients (29% of the total) compared with the Los Angeles group who could have been discharged on oral treatment if such an agent had been available. However, by targeting specific patient groups, we found that 71% of these may be suitable for earlier discharge.

It is interesting that in a number of cases there was a very prolonged stay after patients fulfilled IV to oral switch criteria. Possible contributors to a delay in discharge include occasional inappropriate antimicrobial use, subsequent development of other hospital acquired infection or new pathology, and logistic community or social issues.

Our study focused on identifying patients who met criteria for IV to oral switch, and discharge, rather than those who were actually discharged. Patients were not felt to be eligible for IV to oral antibiotic switch if they did not meet the predetermined IV to oral switch criteria (tables 1 and 2). In some settings a clinical decision may be made to switch to oral therapy even in the presence of nasogastric suction, ileus or bowel obstruction; in which case more patients could potentially be switched. However for the purposes of this study our criteria reflected current guidelines and practice in our hospital.

Pragmatically, a 24 hour observation period between IV-oral switch and discharge is preferred by many clinicians. This is the minimum time period implicit in the criteria used in this study. In practice it is quite possible that discharge would be earlier. However, we suggest that prospective studies should now be carried out to find out if our predictions are borne out in practice. Ideally this would be a randomised controlled trial to compare outcomes between inpatient and outpatient treatment. Unfortunately it is likely that recruitment to this type of study would present problems and patient choice may dictate a significant preference for home treatment. Again, this is an area that needs to be explored systematically. We also suggest that a comparison is made with other options such as an ambulatory IV service. While ambulatory IV therapy retains the disadvantages of IV line use, it may be a cost-effective approach in some settings [19].

Targeted IV to oral switch represents a significant opportunity to improve patient care and reduce the risks and costs associated with hospitalisation. It clearly indicates that different approaches to the management of clinical infection need to be considered. The recent availability of new antimicrobials makes implementation of this approach possible and provides an opportunity to gather an appropriate level of good evidence for the use of oral agents in this setting.

## Conclusion

The need for glycopeptide therapy for infections caused by antibiotic resistant gram positive bacteria has a significant impact on length of stay. Effective targeting of oral antimicrobials could reduce the need for IV access, allow outpatient treatment and thus reduce length of stay. Evidence from these data suggests that this could be effectively targeted to particular groups of patients that can be identified at the start of treatment.

## Competing interests

This investigator-initiated study was funded by a grant from Pfizer.

Pfizer also financed the manuscript publication.

## Authors' contributions

BDF, AJ, AH and KBB conceived and designed this study. ST, BDF, MR and KBB performed the data analysis. ST collected the data. KBB and BDF supervised the study. ST, KBB, BDF and MD were involved in preparation of this manuscript. All authors read and approved the final manuscript.

## Pre-publication history

The pre-publication history for this paper can be accessed here:


